# Estimating Lion Abundance using N-mixture Models for Social Species

**DOI:** 10.1038/srep35920

**Published:** 2016-10-27

**Authors:** Jerrold L. Belant, Florent Bled, Clay M. Wilton, Robert Fyumagwa, Stanslaus B. Mwampeta, Dean E. Beyer

**Affiliations:** 1 Carnivore Ecology Laboratory, Forest and Wildlife Research Center, Mississippi State University, Mississippi State, Mississippi, United States of America; 2 Tanzania Wildlife Research Institute, Arusha, United Republic of Tanzania; 3 Michigan Department of Natural Resources, Marquette, Michigan, United States of America

## Abstract

Declining populations of large carnivores worldwide, and the complexities of managing human-carnivore conflicts, require accurate population estimates of large carnivores to promote their long-term persistence through well-informed management We used N-mixture models to estimate lion (*Panthera leo*) abundance from call-in and track surveys in southeastern Serengeti National Park, Tanzania. Because of potential habituation to broadcasted calls and social behavior, we developed a hierarchical observation process within the N-mixture model conditioning lion detectability on their group response to call-ins and individual detection probabilities. We estimated 270 lions (95% credible interval = 170–551) using call-ins but were unable to estimate lion abundance from track data. We found a weak negative relationship between predicted track density and predicted lion abundance from the call-in surveys. Luminosity was negatively correlated with individual detection probability during call-in surveys. Lion abundance and track density were influenced by landcover, but direction of the corresponding effects were undetermined. N-mixture models allowed us to incorporate multiple parameters (e.g., landcover, luminosity, observer effect) influencing lion abundance and probability of detection directly into abundance estimates. We suggest that N-mixture models employing a hierarchical observation process can be used to estimate abundance of other social, herding, and grouping species.

A dramatic decline in the global conservation status of carnivores has occurred, with more than 20% of species moving at least one IUCN Red List category closer to extinction since 1975^1^. This trend is exacerbated for the world’s largest carnivore species (>15 kg body mass), with more than half reportedly threatened with extinction and about 80% experiencing population declines^2^. Dominant factors causing these declines include geopolitical events (e.g., collapse of the Soviet Union), natural resource exploitation, low social tolerance, and persecution^1^,^2^. Effects of large carnivore declines can be extreme, including increases in herbivore abundance^3^ or mesopredator release^4^, facilitating trophic cascades.

Currently listed by the IUCN as Vulnerable to extinction, the African lion (*Panthera leo*) population has purportedly declined 43% from 1993 to 2014^5^, with greatest declines in West and Central Africa^6^, to an estimated 20,000–35,000 individuals worldwide^5^,^7^. Causes of lion population decline are complex and may vary regionally, with land use change, illegal killing, and prey depletion being the greatest threats to lion population viability^5^,^7^. In addition, persecution of lions through retaliatory killing^8^,^9^, poorly-regulated sport hunting^10^, and demand for traditional medicines^11^ may be important drivers of lion population viability.

Effective management of lions and other large carnivores requires accurate estimates of population sizes and trends to establish harvest regulations, accurately assess conservation status, and understand the effects of dynamic anthropic and environmental conditions. Numerous techniques have been developed to estimate abundance of lions including individual counts^12^, distance sampling^13^, mark-recapture^14^, call-in surveys^15^, camera surveys^16^,^17^, and track counts^18^, with individual counts, call-in surveys, and track counts most commonly used. The accuracy and precision of survey techniques varies and their application has led to scientific debates [e.g. refs 19 and 20] with potentially important implications for species conservation. An important limitation of previous studies is that few have incorporated a repeated design employing spatial or temporal replicates to estimate the probability of detecting individuals to account for lions (or their sign) that were present but not observed. A notable exception was Durant *et al*.^13^ who used a sightability function in distance sampling to correct for estimated lion abundance in Serengeti National Park, Tanzania. Other survey design features that may improve abundance estimates include consideration of the variation in observer’s ability to detect individuals^21^, and incorporation of environmental factors (e.g., prey abundance, luminosity) beyond land cover class [e.g. ref. 15].

*N*-mixture models are a class of models which allow for estimating animal abundance from spatially-replicated data [e.g. ref. 22], and have been demonstrated to be robust across diverse taxa [e.g. refs 23 and 24]. The abundance of clouded leopards (*Neofelis diardi*) has been estimated using *N*-mixture models^25^, but to our knowledge these models have not been applied to lions. Our primary objective was to estimate lion abundance in a portion of Serengeti National Park using *N*-mixture models with data from repeated call-in and track surveys. Our secondary objective was to identify ecological and observation process variables that influence abundance estimation.

## Results

Goodness-of-fit of the selected models for both call-in surveys and track data was good, with Bayesian p-values of 0.4 and 0.52 respectively (see Supplementary Figs S1 and S2).

**Figure 1:**
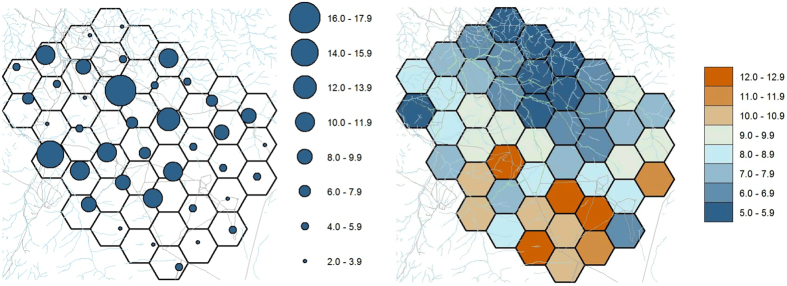
Predicted number of lions occurring at call-in sites (left panel) and predicted number of tracks/km (right panel), southeastern Serengeti National Park, Tanzania, September–November 2015.

**Figure 2:**
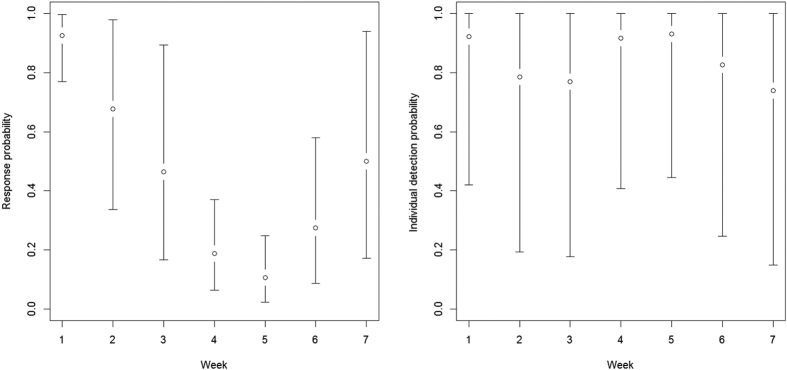
Weekly probability (and 95% confidence intervals) of response (left) and individual (right) detection probability of lions to broadcasted vocalizations during call-in survey, southeastern Serengeti National Park, Tanzania, September–November 2015.

Using call-in surveys, we estimated an abundance of 270 lions over the sampled area (median = 242; 95% credible interval = 170–551). Estimated number of lions at individual call-in sites ranged from 2.0 to 17.9 (Fig. 1). Assuming the area sampled reflected our study area, lion density was 14.4 lions/100 km^2^. The probability of lion groups responding to a call appeared to vary across weeks, with point estimates declining from 0.93 in week 1 to 0.11 in week 5, then increasing to 0.50 in week 7 (Fig. 2). In contrast, probability of detection conditional on group response across weeks was less variable (0.74–0.92). We detected a negative effect of lunar illumination on lion individual detectability but no determinable effect of land cover on abundance (Table 1); other covariates were not included in the final model.

We detected 456 lion track occurrences overall; the total number of tracks detected varied among routes (0–129) and weeks (52–91). The predicted number of tracks/km in 40 km^2^ cells ranged from 5.0 to 12.9 (Fig. 1). Mean total number of tracks estimated to occur across all roads within the study area was 1366 (median = 608; 95% credible interval = 370–7335). Land covers were included in the final model but had no determinable effect on track density (Table 1). We found a weak negative relationship between predicted track density and predicted lion abundance from the call-in surveys across 40 km^2^ cells (*R*^*2*^ = 0.113, P = 0.012) and were therefore unable to estimate lion abundance using track density estimates.

## Discussion

We provide the first estimate of lion abundance using *N*-mixture models and the first to incorporate a hierarchical observation process specifically designed to account for the behaviors of social species by integrating both group responses and individual detectability. Though direct comparisons of lion abundance estimates in SNP are precluded due to differences in study areas and methodologies, our estimate of 270 lions (14.4 lions/100 km^2^) from call-in surveys compares favorably with previous estimates. Using distance sampling in a 2,306 km^2^ area of SNP and Ngorongoro Conservation Area which included our study area, estimated lion abundance in September 2002 was 314 individuals (95% CI = 136–725), or 13.6 lions/100 km^2^ 13. Again using distance sampling in a 2,492 km^2^ area in October 2005 which also included our study area, Durant *et al*.^13^ estimated 247 lions (95% CI = 137–444), a density of 9.9 individuals/100 km^2^. Areas surveyed by Durant *et al*.^13^ outside our study area reportedly contained few lions; thus, lion density in the area surveyed in common by us and Durant *et al*.^13^ was likely more similar than their reported overall densities. We acknowledge that our estimate of lion density may be biased slightly high due to potential dependence between adjacent call-in sites resulting in double-counting of some individuals. However, attracting the same lions to adjacent sites is unlikely based on previous work^15^ which suggests lions do not typically approach from greater than 3 km based on the duration and intensity of our broadcasted vocalizations.

Using random encounter models from remote camera imagery in a portion of our study area, Cusack *et al*.^16^ estimated 14.4 females/100 km^2^ in grassland and 21.3 females/100 km^2^ in woodland. These estimates are greater than female densities in grassland (12.4/100 km^2^) and woodland (14.2/100 km^2^) using their reference population of known individuals^16^. Overall densities from remote cameras may be biased even higher as subadults and adult male lions were not estimated. Total lion abundance in this study area is largely static^13^,^26^, with episodic changes occurring only every 10–20 years^26^. That lion density in our study that included adult males and subadults was 2.0 individuals/100 km^2^ greater than the density of females in the reference population suggests our estimate is reasonable.

Lion individual detectability during call-ins decreased with increasing luminosity. Lions are largely nocturnal predators^27^ but less successful at capturing wild prey during nights with high luminosity^18^,^28^. Activity of many prey species increases with increasing lunar illumination, consistent with the hypothesis that increasing luminosity facilitates detection of predators by prey^29^. Lions in our study may have had reduced movements during nights with high luminosity due to their increased visibility by prey, which could reduce their probability of approaching calls.

Numerous authors have suggested lions habituate to broadcasted calls [e.g. ref. 15]. We demonstrated apparent rapid habituation to broadcasted calls, with lion response declining dramatically after the first week of surveys, in addition to varying in response to lunar illuminosity. Though we were able to estimate lion abundance, reducing the habituation response would reduce the effects of zero-inflation in our models and improve overall precision. Alternating call sequences and/or locations across weeks or increasing the interval between sessions could reduce habituation and warrants further investigation.

Land cover was included in our final models of lion abundance and track density, but we were unable to determine the corresponding direction of response. Midlane *et al*.^30^ found that stratification by land cover did not improve their estimate of lion abundance. Though habitat features influence lion distribution^31^, it is largely through improved accessibility to prey and at a finer resolution^32^ than used in this study. Using landscape metrics more strongly related to lion resource use and monitoring prey distributions or abundance could improve performance of our models.

We found poor correlation between predicted lion abundance and track density (tracks/km). Tracks have been used to estimate abundance of lions and other large carnivores [e.g. ref. 18]. Our lack of an observed relationship could be a consequence of small resolution of cells used, not identifying appropriate covariates to explain track density, or both. More practically, it could be a consequence of our inability to identify individuals from tracks. Karanth *et al*.^33^ recommended collecting track data from all four paws on good substrate for individual identification; however, we did not measure all tracks due to varying quality of substrate and uneven road surfaces. Though we attempted to discern individuals, overlapping measurements of tracks made individual identification challenging. Therefore it is possible that we both overestimated and underestimated the number of lions based on tracks across survey routes and weeks. Direct counts of animals through observation are typically preferred over indirect measures such as tracks for abundance estimation^34^. Though different field and modeling techniques were used, we agree with Midlane *et al*.^30^ who also found call-in surveys more suitable than track surveys for estimating lion abundance.

There are several advantages in using our approach with call-in surveys to estimate lion abundance. In contrast to distance sampling, call-in surveys are effective in savanna and forested systems. Our repeated call-in surveys also can be conducted in a shorter time period than some other methods (e.g., remote cameras)^16^, facilitating assumptions of geographic and demographic closure. In contrast to previous surveys, use of *N*-mixture models allowed us to account for observer variation which can have a strong influence on species detection [e.g. ref. 35]. Further, repeated surveys allowed us to account for temporal variation in environmental conditions (e.g., luminosity) not previously considered but known to influence lion behavior^29^ and thus, detectability. We encourage additional examination of environmental covariates that could influence detection and occupancy of species or their sign. Finally, our hierarchical detection process provides the first effort to account for the sociality of lions, specifically that individual pride members approaching our call-in sites or deposition of their tracks are not independent. We suggest that wildlife biologists use *N*-mixture models incorporating a hierarchical observation process to estimate abundance of other social, herding, and grouping species (e.g., ungulates, birds, fish).

## Materials and Methods

All sampling methods complied with guidelines established by the American Society of Mammalogists^36^ and field techniques were approved by the Institutional Animal Care and Use Committee protocol (approval 16–030) at Mississippi State University. Sampling locations and procedures were approved by the Tanzania Wildlife Research Institute, Tanzania National Parks, and the Commission for Science and Technology (permit 2015–198-NA-2015–166). Sampling procedures involved observation of protected species (i.e., *P. leo*).

### Study Area

**Figure 3:**
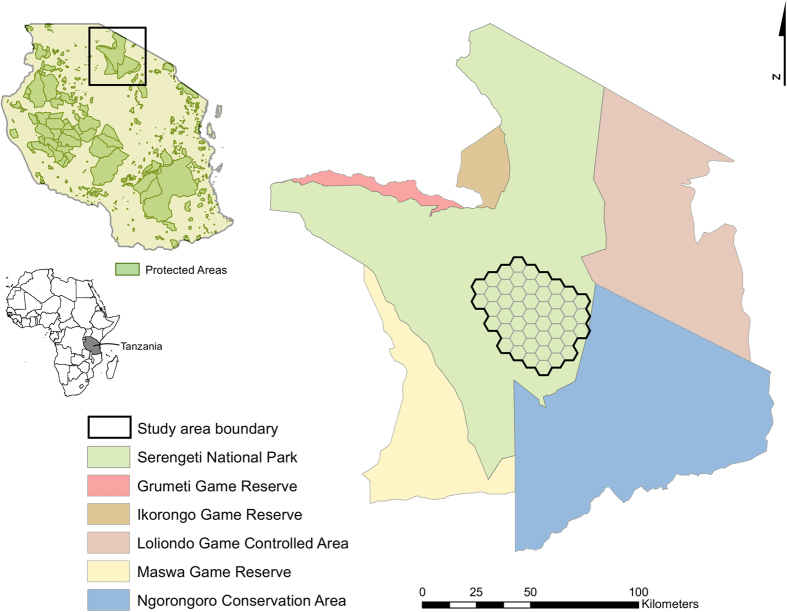
Location of study area to estimate lion abundance, southeastern Serengeti National Park, Tanzania, September–November 2015. Maps created in ArcMap (version 10.1; www.esri.com).

We conducted this study in a 1,880 km^2^ area in southeastern Serengeti National Park, Tanzania (Fig. 3). Most rainfall in this savanna system occurs during November–May, increasing from the southeast to northwest^37^. Vegetation response to rainfall results in short-grass savanna in the southeast, transitioning to tall-grass savanna before becoming woodland in the northwest part of the study area^38^. Woody vegetation is most extensive along rivers and rock outcrops (kopjes) occur throughout the study area.

### Call-in survey

**Figure 4:**
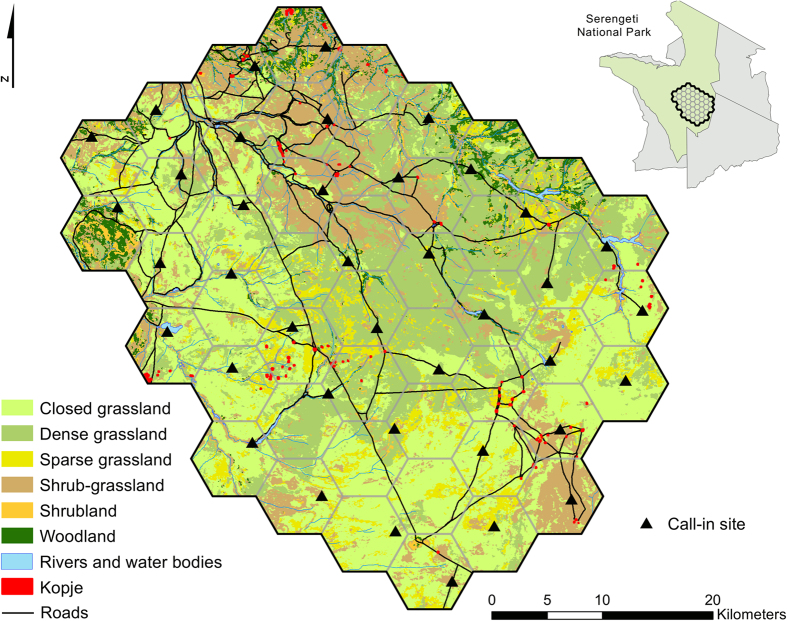
Locations of call-in sites to estimate lion abundance, southeastern Serengeti National Park, Tanzania, September–November 2015. Maps created in ArcMap (version 10.1; www.esri.com).

We established 39 call-in sites with spacing of about 6 km between sites (Fig. 4). Lion movements and their ability to detect broadcasted vocalizations from >3 km distant^37^, could result in overestimates of abundance through double counting some individuals. However, we suggest abundance estimates to represent the entire study area, with minimal overlap between sites, based on the length and intensity of call-ins [see ref. 15]. Around each site, we created a 3-km radius (28.27 km^2^) buffer and used GIS to estimate the percentage of each land cover, km of rivers or stream, km of roads, and number of kopjes. We obtained GIS layers of landscape attributes from the Serengeti-Mara database managed by Tanzania National Parks and Frankfurt Zoological Society (http://www.serengetidata.org/). To facilitate modeling, we combined existing land covers into 6 classes including sparse grassland, closed grassland, dense grassland, shrub-grassland, shrubland, and woodland [see ref. 39].

Using two crews, we conducted the call-in survey for 7 weeks beginning mid-September 2015, broadcasting at 7 or 8 sites each night and completing the 39 sites each week. We began broadcasts at 1900 h when lions increase movements^40^. We calculated luminosity values for each night using the R package lunar^41^.

**Figure 5:**
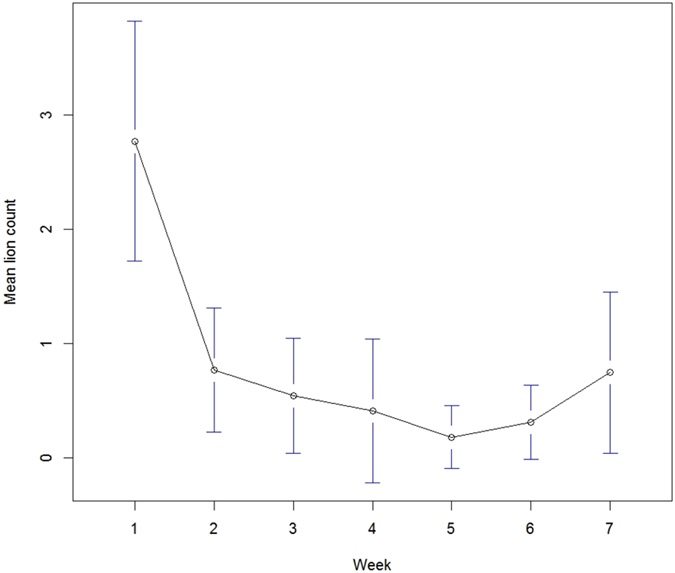
Weekly mean (and 95% confidence intervals) number of lions detected during call-in survey, southeastern Serengeti National Park, Tanzania, September–November 2015.

We used a digital recording comprised of a single female lion roar, wildebeest (*Connochaetes taurinus*) in distress, and spotted hyena (*Crocuta crocuta*) whoop call; vocalizations previously demonstrated successful in attracting lions^15^. We broadcasted vocalizations at each site for 70 min^42^, playing calls for 10 min, followed by a 5-min pause, and then repeating this pattern 5 times for 70 min. We selected 70 min as lions can take up to 70 min to approach and be detected^15^. Each 10-min broadcast started with 37 s of a single female lion roar, followed by 2 min 5 s of a wildebeest in distress, and 38 s of a spotted hyena whoop call; this sequence was repeated 3 times. We broadcasted calls at up to 116 dB using a commercial game calling system (Foxpro Inc., Lewistown, Pennsylvania, USA). We used 4 speakers mounted at 90 degree intervals on the roof of the vehicle (about 2.4 m above ground). As the amperage required by the speaker system was too great for the vehicle battery without running the engine, we alternated broadcasts between opposing pairs of speakers midway through each 10-min broadcast. We alternated call-in sites surveyed by each crew each week to account for variation in detection between crews. Because we detected a decrease in the number of lions at call-in sites across weeks 1–5 (Fig. 5), during weeks 6 and 7 we used buffalo (*Syncerus caffer*^15^,^43^) distress calls instead of wildebeest calls at some sites.

Through a vehicle roof hatch, we counted and recorded the number of lions throughout the broadcast using a spotlight with red filter (Model EF170CC; Lightforce USA, Inc., Orofino, Idaho, USA) and forward-looking infrared monocular (FLIR Scout TS24; Tactical Night Vision Company, Redlands, California, USA). We used a red filter as we noticed some aversion by lions to the unfiltered light during preliminary call-ins conducted before the survey. We used the maximum number of lions detected at each site during each 70-min call-in to estimate abundance.

### Track survey

**Figure 6:**
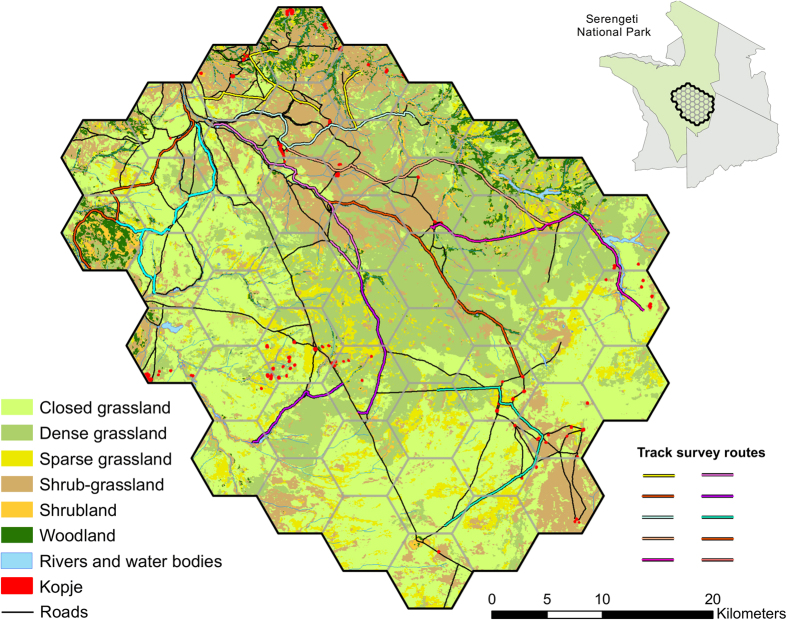
Location of track survey routes to estimate lion abundance, southeastern Serengeti National Park, Tanzania, September–November 2015. Maps created in ArcMap (version 10.1; www.esri.com).

We established 10 transects on roads,each about 25 km long (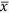 = 25.3 km, σ = 1.12 km, 253.1 km total; Fig. 6). Distance of roads surveyed in cells ranged from 0 to 20 km. Though track substrate can influence track deposition; road substrates in our study area were previously categorized as clay only^18^. We surveyed each transect once each week for 7 weeks. We cleared tracks on routes the evening before surveying them (typically 1700–1830 hrs) using a tire drag pulled behind each vehicle. Each of the two track survey crews consisted of a driver and an experienced tracker positioned on the hood of the vehicle. Surveys began at about 0700 hr and were typically completed before 1200 hr to reduce the negative effects of direct sunlight on detecting tracks. Each crew travelled along routes at speeds up to 10 km/hour.

When we detected lion tracks, we identified the number of individuals using track size, juxtaposition, and direction of travel; measured the length and width of a representative track of each individual; and took an image of each for reference. Tracks that were located further along the respective routes were counted as new individuals if it could not be determined using our criteria and images that tracks were from the same individuals identified previously. Leopards (*Panthera pardus*) are rare in our grassland-dominated study area. We distinguished the occasional leopard track from lion tracks using track size, shape of pads, group size (leopards are typically solitary), and location (leopards largely restrict movements to wooded riparian areas). We discarded any track that could not reliably be identified as lion. As with call-in surveys, we alternated the routes crews surveyed each week to account for variation in detection between crews. As track surveys were conducted during the same 7 weeks as call-in surveys, we ensured track routes were not sampled within 24 hrs of overlapping call-in sites.

To develop estimates of track densities and compare these densities to lion abundance for the same area from the call-in survey, we established a grid of 47, 40-km^2^ cells (Fig. 6). For each cell, we determined the area of each land cover, km of rivers and streams, km of roads, and number of kopjes as described for call-in site buffers.

### Statistical analyses

We used a similar approach to model abundance for call-in responses (number of individuals at a call-in site) and track counts (number of tracks/km/cell). To account for imperfect detection in our datasets, we used a hierarchical modeling approach. We modeled abundance (call-ins) and track density (tracks) using *N*-mixture models^22^, conceptually similar to the generalized N-mixture model developed by Chandler *et al*.^44^. For both datatypes, we modified this model to describe relationships between our abundance process and our environmental covariates (land cover, km of river, km of road, number of kopjes), as well as between our detection process and the observers’ abilities. N-mixture models commonly assume closure in the studied population. While this assumption might not be fully met because of potential temporary immigration and emigration from our study site, our choice of seven consecutive weekly temporal replicates provides a good approximation to meet this assumption for a lion population. The “true” ecological state *N*_*i*_ describing abundance (number of individuals in the area of influence of our call-in sites), or track density (number of tracks per kilometer of road) in site *i* was defined as a Poisson random variable, with an expected value λ_i_. A site corresponded to a cell for track analysis, and a call-in site for the call-in survey. We modeled the expected value of the Poisson distribution as a linear expression of an intercept (*a*_*0*_), our environmental covariates, and a random site effect (*ε*_*i*_) on the log-scale such as:


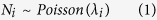



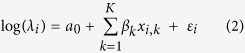


where *x*_*i,k*_ denotes to the value of scaled environmental covariate *k* at site *i*, and *β*_*k*_ the corresponding slope. Because we detected at least one individual at each site, indicating a non-null population at every site, to speed convergence time, we bounded log(λ_i_) to vary between 0.1 and 10.

To account for detectability imperfections, we modeled the count process *y*_*it*_ in cell *i* during week *t* conditionally on the true abundance, such as:


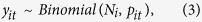


where *p*_*it*_ is the individual detection probability in cell *i* during week *t.* For analysis of track data, we allowed detection probability *p*_*it*_ to vary among sites and weeks depending on observers, and used a logistic linear model of the form:


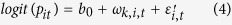


with *b*_*0*_ an intercept, a *ω*_*k,i,t*_ random observer-effect for the observer *k* present in cell *i* during week *t,* and a random cell-week effect (*ε*′_*i,t*_).

The analysis of call-in data required more detailed modeling of the observation process. First, lion’s responses suggested habituation to broadcasted calls (Fig. 5), with point estimates of detection probabilities generally declining across weeks. Second, because lions are social, groups rather than individuals often respond. Therefore, we used two levels for our detection probability where 1) groups can respond to a call if any individual of the group responds and 2) if a group responds, each individual is potentially available for detection. The approach we used to model this hierarchical relationship can be assimilated to a zero-inflated binomial distribution where the detection probability is modeled as:


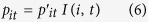


with *I*(*i,t*) an indicator function following a Bernoulli distribution with mean *p*″_*t*_. The probability *p*″_*t*_ can be described as the probability that individuals will respond to a call during week *t*. If an individual responds to a call (i.e., *I*(*i,t*) = 1), it becomes available for detection at the call-in site with an individual detection probability *p*′_*it*_, effectively conditioning the global detection probability *p*_*it*_ at site *i* during week *t* on the initial response to calls. We modeled the individual detection probability *p*′_*it*_ as a linear combination of an intercept, a lunar illumination effect, a week effect, as well as a random observer effect and a random site-week effect on the logit scale. N-mixture models usually assume independent individual detection. Because of the group response of social species, this assumption would be violated using a regular N-mixture model, but the approach we used allowed us to account for both group and individual detectabilities.

Finally, we estimated the population size over the sampled area, by summing the estimated abundance from each site, assuming there was no overlap between areas covered by call-in sites. We derived predicted values of abundance and track density for each hexagonal cell in our study area, based on the estimated parameters from the above analyses, and the corresponding environmental predictive covariates selected in the model. We used these predictions to evaluate the correlation between estimated abundance and track density in our study area with a simple linear regression.

We implemented models for track density data and for call-in counts using the program WinBUGS (see Supplementary Files S3). We used non-informative priors for each parameter. We ran 3 chains of 100,000 iterations after a 100,000 burn-in with a thinning of 10, and monitored convergence by visual inspection of the MCMC chains and using the Gelman-Rubin convergence statistic 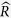. We performed model selection using the variable selection process for regression models^45^,^46^. We included in the final models variables that were selected at least 10% of the time and re-ran the analyses with these covariates to provide more precise estimates of the corresponding parameters. We assessed goodness-of-fit of the selected models based on their corresponding Bayesian p-values, with values close to 0.5 indicating fit and values close to 0 or 1 indicating lack of fit. We present average estimated abundance at call-in sites and track density per cell, as well as corresponding detection probabilities with 95% confidence intervals.

## Additional Information

**How to cite this article**: Belant, J. L. *et al*. Estimating Lion Abundance using N-mixture Models for Social Species. *Sci. Rep.* **6**, 35920; doi: 10.1038/srep35920 (2016).

**Publisher’s note:** Springer Nature remains neutral with regard to jurisdictional claims in published maps and institutional affiliations.

## Supplementary Material

Supplementary Information

Supplementary Table S1

Supplementary Table S2

Supplementary Table S3

Supplementary Table S4

## Figures and Tables

**Table 1:** Covariates influencing lion abundance and track density, southeastern Serengeti National Park, Tanzania, September–November 2015.

Technique	Covariate	Mean	Credible Interval	
			2.5%	97.5%
Call-in	Lunar illumination	−3.99	−8.65	−0.36
	Closed grassland	−1.13	−11.84	9.27
	Woodland	−2.75	−18.74	13.63
	Sparse grassland	−0.65	−15.82	14.67
	Shrubland	1.83	−16.93	20.51
	Dense grassland	2.82	−8.06	13.68
	Shrub-grassland	−0.47	−11.41	10.2
Track	Closed grassland	1.01	−2.07	3.98
	Woodland	−2.65	−12.35	7.49
	Sparse grassland	5.31	−5.3	15.76
	Shrubland	−0.16	−16.9	15.94
