# Supplementary material for: Estimating Lion Abundance using N-mixture Models for Social Species
Source: Sci Rep. 2016 Oct 27;6:35920. doi: 10.1038/srep35920 (PMC5082374; doi:10.1038/srep35920)
Supplement: Supplementary Information [file srep35920-s1.doc]

Estimating Lion Abundance using N-mixture Models for Social Species

Jerrold L. Belant^1*^, Florent Bled^1^, Clay M. Wilton^1^, Robert Fyumagwa^2^, Stanslaus B. Mwampeta^1^, Dean E. Beyer, Jr.^3^

^1^ Carnivore Ecology Laboratory, Forest and Wildlife Research Center, Mississippi State University, Mississippi State, Mississippi, United States of America

^2^ Tanzania Wildlife Research Institute, Arusha, United Republic of Tanzania

^3^ Michigan Department of Natural Resources, Marquette, Michigan, United States of America

^*^ Corresponding author; E-mail: [j.belant@msstate.edu](mailto:j.belant@msstate.edu)

**S1 Figure.** Goodness of fit results for call-in survey, Serengeti National Park, Tanzania, September–November 2015.


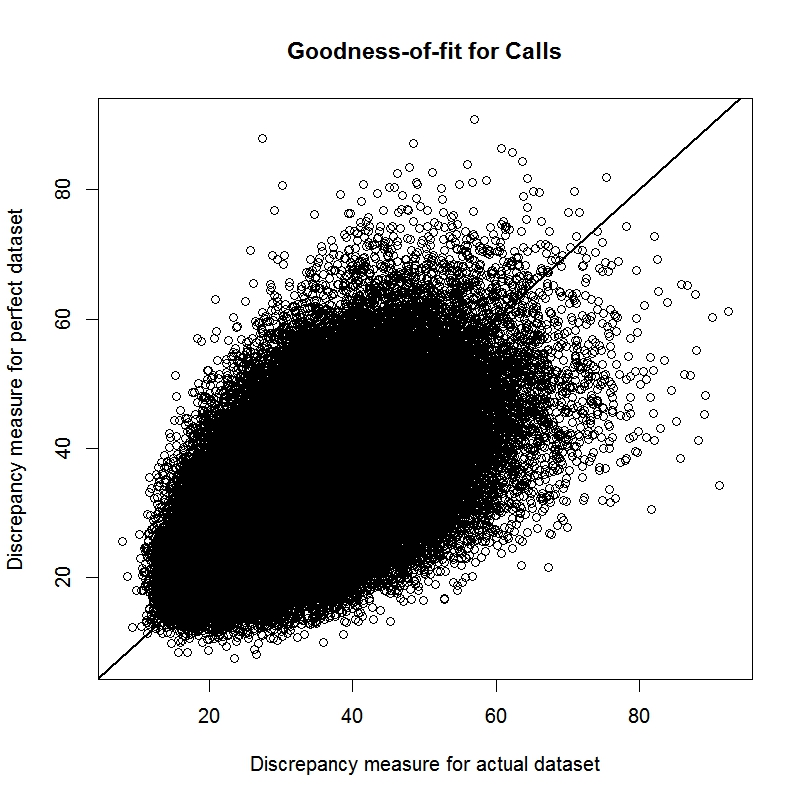


**S2 Figure.** Goodness of fit results for track survey, Serengeti National Park, Tanzania, September–November 2015.


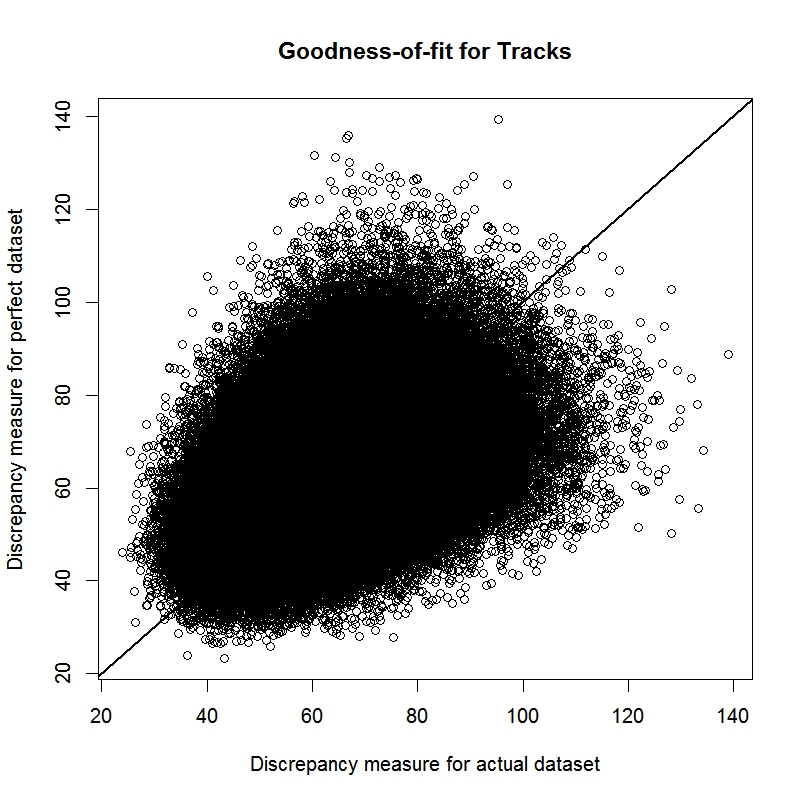


**S3 File. Statistical analysis code.** WinBUGS code to estimate lion and track abundance, Serengeti National Park, Tanzania, September–November 2015.

model {

###### CALLS ########

## Priors

# Ecological process

intercept.abun.c ~ dnorm(0, 0.01)

m.rdl.c ~ dnorm(0, 0.01)

m.rrl.c ~ dnorm(0, 0.01)

m.kc.c ~ dnorm(0, 0.01)

m.cgd.c ~ dnorm(0, 0.01)

m.dgd.c ~ dnorm(0, 0.01)

m.fwd.c ~ dnorm(0, 0.01)

m.sd.c ~ dnorm(0, 0.01)

m.osgd.c ~ dnorm(0, 0.01)

m.sgd.c ~ dnorm(0, 0.01)

# Observation process

int.detect.c ~ dnorm(0, 0.01)

m.week.c ~ dnorm(0, 0.01)

m.lunar.c ~ dnorm(0, 0.01)

# Random effects

for (i in 1:n.site.c) { # Loop over sites

u.abun.c[i] ~ dnorm(0, tau.N.c)

for (t in 1:n.week) { # Loop over surveys

u.p.c[i,t] ~ dnorm(0, tau.p.c)

}

}

for(k in 1:n.obs.c){

u.obs.c[k] ~ dnorm(0, tau.obs.c)

}

tau.N.c <- pow(sigma.N.c, -2)

sigma.N.c ~ dunif(0, 5)

tau.p.c <- pow(sigma.p.c, -2)

sigma.p.c ~ dunif(0, 5)

tau.obs.c <- pow(sigma.obs.c, -2)

sigma.obs.c ~ dunif(0, 5)

# Model selection

for (m in 1:n.param.c){

mod.sel.c[m]~dbern(p.sel.c)

}

p.sel.c~dbeta(2,8)

## State process

for (i in 1:n.site.c) { # Loop over sites

N.c[i] ~ dpois(lambda.c[i])

log(lambda.c[i]) <- max(0.1,min(loglam.c[i],10))

loglam.c[i] <- intercept.abun.c + u.abun.c[i]

+ mod.sel.c[1]*m.rdl.c*Road_Length.c[i]

+ mod.sel.c[2]*m.rrl.c*River_Length.c[i]

+ mod.sel.c[3]*m.kc.c*Kopje_Count.c[i]

+ mod.sel.c[4]*m.cgd.c*Closed_Grassland.c[i]

+ mod.sel.c[5]*m.dgd.c*Dense_Grassland.c[i]

+ mod.sel.c[6]*m.fwd.c*Forest_Woodland.c[i]

+ mod.sel.c[7]*m.sd.c*Shrubland.c[i]

+ mod.sel.c[8]*m.osgd.c*Open_Sparse_Grassland.c[i]

+ mod.sel.c[9]*m.sgd.c*Shrubbed_Grassland.c[i]

## Observation process

for (t in 1:n.week) { # Loop over surveys

y.c[i,t] ~ dbin(p.c[i,t], N.c[i])

p.c[i,t] <- indic.p.c[i,t] * ( 1 / (1 + exp( -1 * ( int.detect.c + u.obs.c[obs.ID.c[i,t]] + mod.sel.c[10]*m.week.c*week[t] + u.p.c[i,t] + mod.sel.c[11]*m.lunar.c*lunar.illumination[t] ))))

}

}

## Availability for detection Indicator (0s indic.p)

for (t in 1:n.week) { # Loop over surveys

p.indic.c[t] ~ dunif(0,1)

for (i in 1:n.site.c) { # Loop over sites

indic.p.c[i,t] ~ dbern(p.indic.c[t])

}

}

## Derived quantities

totalN.c <- sum(N.c[]) # Population size over all R sites

logsigma.N.c <- log(sigma.N.c)

logsigma.p.c <- log(sigma.p.c)

logsigma.obs.c <- log(sigma.obs.c)

# GOF

for (i in 1:n.site.c) { # Loop over sites

for (t in 1:n.week) { # Loop over surveys

# Compute fit statistics for observed data

eval.c[i,t]<-p.c[i,t]*N.c[i]

E.c[i,t]<- pow((y.c[i,t]-eval.c[i,t]),2)/(eval.c[i,t]+0.5)

# Generate replicate data and compute fit stats for them

Y.new.c[i,t] ~ dbin(p.c[i,t],N.c[i])

E.new.c[i,t]<- pow((Y.new.c[i,t]-eval.c[i,t]),2)/(eval.c[i,t]+0.5)

}

}

fit.c<-sum(E.c[,])

fit.new.c<-sum(E.new.c[,])

# Detection probability

for (t in 1:n.week) { # Loop over surveys

p.week.c[t] <- (1 / (1 + exp( -1 * ( int.detect.c + 0.5*sigma.obs.c*sigma.obs.c + mod.sel.c[10]*m.week.c*week[t] + 0.5*sigma.p.c*sigma.p.c + mod.sel.c[11]*m.lunar.c*lunar.illumination[t] ))))

}

# Prediction for hexagonal cells

for (i in 1:n.cell) { # Loop over cells

N.cell.c[i] ~ dpois(lambda.cell.c[i])

log(lambda.cell.c[i]) <- max(0.1,min(loglam.cell.c[i],10))

loglam.cell.c[i] <- intercept.abun.c + 0.5*sigma.N.c*sigma.N.c

+ mod.sel.c[1]*m.rdl.c*Road_Length.cell[i]

+ mod.sel.c[2]*m.rrl.c*River_Length.cell[i]

+ mod.sel.c[3]*m.kc.c*Kopje_Count.cell[i]

+ mod.sel.c[4]*m.cgd.c*Closed_Grassland.cell[i]

+ mod.sel.c[5]*m.dgd.c*Dense_Grassland.cell[i]

+ mod.sel.c[6]*m.fwd.c*Forest_Woodland.cell[i]

+ mod.sel.c[7]*m.sd.c*Shrubland.cell[i]

+ mod.sel.c[8]*m.osgd.c*Open_Sparse_Grassland.cell[i]

+ mod.sel.c[9]*m.sgd.c*Shrubbed_Grassland.cell[i]

}

####### TRACKS ########

## Priors

# Ecological process

intercept.abun.t ~ dnorm(0, 0.01)

m.rdl.t ~ dnorm(0, 0.01)

m.rrl.t ~ dnorm(0, 0.01)

m.kc.t ~ dnorm(0, 0.01)

m.cgd.t ~ dnorm(0, 0.01)

m.dgd.t ~ dnorm(0, 0.01)

m.fwd.t ~ dnorm(0, 0.01)

m.sd.t ~ dnorm(0, 0.01)

m.osgd.t ~ dnorm(0, 0.01)

m.sgd.t ~ dnorm(0, 0.01)

# Observation process

int.detect.t ~ dnorm(0, 0.01)

m.week.t ~ dnorm(0, 0.01)

# Random effects

for (i in 1:n.site.t) { # Loop over sites

u.abun.t[i] ~ dnorm(0, tau.N.t)

for (t in 1:n.week) { # Loop over surveys

u.p.t[i,t] ~ dnorm(0, tau.p.t)

}

}

for(k in 1:n.obs.t){

u.obs.t[k] ~ dnorm(0, tau.obs.t)

}

tau.N.t <- pow(sigma.N.t, -2)

sigma.N.t ~ dunif(0, 5)

tau.p.t <- pow(sigma.p.t, -2)

sigma.p.t ~ dunif(0, 5)

tau.obs.t <- pow(sigma.obs.t, -2)

sigma.obs.t ~ dunif(0, 10)

# Model selection

for (m in 1:n.param.t){

mod.sel.t[m]~dbern(p.sel.t)

}

p.sel.t~dbeta(2,8)

## State process

for (i in 1:n.site.t) { # Loop over sites

N.t[i] ~ dpois(lambda.t[i])

log(lambda.t[i]) <- max(0.1,min(loglam.t[i],10))

loglam.t[i] <- intercept.abun.t + u.abun.t[i]

+ log(RouteLength_Km.t[i])

+ mod.sel.t[1]*m.rdl.t*Road_Length_Km.t[i]

+ mod.sel.t[2]*m.rrl.t*River_Length_Km.t[i]

+ mod.sel.t[3]*m.kc.t*Kopje_Count.t[i]

+ mod.sel.t[4]*m.cgd.t*Closed_Grassland.t[i]

+ mod.sel.t[5]*m.dgd.t*Dense_Grassland.t[i]

+ mod.sel.t[6]*m.fwd.t*Forest_Woodland.t[i]

+ mod.sel.t[7]*m.sd.t*Shrubland.t[i]

+ mod.sel.t[8]*m.osgd.t*Open_Sparse_Grassland.t[i]

+ mod.sel.t[9]*m.sgd.t*Shrubbed_Grassland.t[i]

## Observation process

for (t in 1:n.week) { # Loop over surveys

y.t[i,t] ~ dbin(p.t[i,t], N.t[i])

p.t[i,t] <- ( 1 / (1 + exp( -1 * ( int.detect.t + u.obs.t[obs.ID.t[i,t]] + u.p.t[i,t] ))))

}

}

## Derived quantities

totalN.t <- sum(N.t[]) # Population size over all R sites

logsigma.N.t <- log(sigma.N.t)

logsigma.p.t <- log(sigma.p.t)

logsigma.obs.t <- log(sigma.obs.t)

# GOF

for (i in 1:n.site.t) { # Loop over sites

for (t in 1:n.week) { # Loop over surveys

# Compute fit statistics for observed data

eval.t[i,t]<-p.t[i,t]*N.t[i]

E.t[i,t]<- pow((y.t[i,t]-eval.t[i,t]),2)/(eval.t[i,t]+0.5)

# Generate replicate data and compute fit stats for them

Y.new.t[i,t] ~ dbin(p.t[i,t],N.t[i])

E.new.t[i,t]<- pow((Y.new.t[i,t]-eval.t[i,t]),2)/(eval.t[i,t]+0.5)

}

}

fit.t<-sum(E.t[,])

fit.new.t<-sum(E.new.t[,])

# Detection probability

for (t in 1:n.week) { # Loop over surveys

p.week.t[t] <- (1 / (1 + exp( -1 * ( int.detect.t + 0.5*sigma.obs.t*sigma.obs.t + 0.5*sigma.p.t*sigma.p.t ))))

}

# Prediction for hexagonal cells

for (i in 1:n.cell) { # Loop over cells

N.cell.t[i] ~ dpois(lambda.cell.t[i])

log(lambda.cell.t[i]) <- max(0.1,min(loglam.cell.t[i],10))

loglam.cell.t[i] <- intercept.abun.t + 0.5*sigma.N.t*sigma.N.t

+ mod.sel.t[1]*m.rdl.t*Road_Length.cell[i]

+ mod.sel.t[2]*m.rrl.t*River_Length.cell[i]

+ mod.sel.t[3]*m.kc.t*Kopje_Count.cell[i]

+ mod.sel.t[4]*m.cgd.t*Closed_Grassland.cell[i]

+ mod.sel.t[5]*m.dgd.t*Dense_Grassland.cell[i]

+ mod.sel.t[6]*m.fwd.t*Forest_Woodland.cell[i]

+ mod.sel.t[7]*m.sd.t*Shrubland.cell[i]

+ mod.sel.t[8]*m.osgd.t*Open_Sparse_Grassland.cell[i]

+ mod.sel.t[9]*m.sgd.t*Shrubbed_Grassland.cell[i]

}

}
